# Mindfulness-based emotional eating awareness training: taking the emotional out of eating

**DOI:** 10.1007/s40519-019-00667-y

**Published:** 2019-03-11

**Authors:** Paul Lattimore

**Affiliations:** School of Natural Sciences and Psychology, Liverpool John Moores University, Liverpool, L3 3AF UK

**Keywords:** Mindfulness, Emotional eating, Intuitive eating, Inhibitory control, Emotion regulation, Stress

## Abstract

**Purpose:**

Emotional eating is important to study and address because it predicts poor outcome in weight loss interventions. Interventions have only touched the surface in terms of addressing emotional eating. Mindfulness approaches can address emotional eating by modification of emotion regulation and appetitive traits. The current study involved development of an emotional eating-specific mindfulness intervention and assessment of its effect on appetitive traits associated with emotional eating.

**Methods:**

Participants (*n* = 14; age *M* = 29 years; 90% female) completed baseline and end-of-intervention self-report measures of emotional eating, food-cue reactivity, mindfulness, intuitive eating, emotional impulse regulation, stress, and a behavioural measure of inhibitory control. During the 6-week intervention, mindfulness meditation skills were taught weekly embedded in a psycho-educational curriculum about emotional eating.

**Results:**

Paired *t* tests, controlled for type 1 error, revealed significant improvements in food-cue reactivity, intuitive eating, emotional impulse regulation, inhibitory control and stress (*p*s < 0.05; *d*: 0.58–1.54). Changes in emotional eating approached significance (*p* = 0.075, *d* = 0.66).

**Conclusion:**

The intervention purposefully did not focus on weight loss and recruited participants who had self-declared difficulties with emotional eating. The positive outcomes suggest that intervening with mindfulness training before weight loss is attempted has the potential to change psychological factors that underpin overeating and undermine weight loss efforts. The study provides proof of principle as a basis to design a randomized control trial to assess rigorously the effectiveness of the intervention as a precursor to a weight loss intervention.

**Level of evidence:**

Level IV, uncontrolled trial.

## Introduction

Emotional eating is a response to negative emotions: a “tendency to overeat in response to negative emotions, such as anxiety or irritability” [1] (p. 106). More recently, researchers view it as a response to both negative and positive mood [2–4]. Emotional eating in eating disorders may reflect general difficulties with emotion regulation [5]. Emotional eating is linked to physical and psychological problems including: body weight fluctuation [6–8]; disruption of weight loss effort [9, 10]; binge eating [11]; and low mood and depression [12]. Reduction in emotional eating predicts successful weight loss [13]. The emotional eating concept is not as simple as it seems and recent examination of how it is typically measured reveals it may reflect lack of control, learned cue reactivity, and misattribution of negative affect to episodic overeating [4]. In this respect the way emotional eating is currently measured may reflect ‘worry’ or ‘overconcern’ [4] that precedes disinhibited eating, the latter driven by learned hedonic cue-reactivity and weakened inhibitory control [14, 15] especially when intense emotions are active [16]. In the context of obesity, emotional eating is important to study and address through intervention because it predicts poor outcome in weight loss interventions; non-emotional eaters are twice as likely to achieve the 10% weight reduction goal of standard behavioural weight loss treatment [10].

It is highly likely that the emotional eating construct is multifaceted and context dependent, a construct that cannot be fully captured using unidimensional measures such as the Dutch Eating Behaviour or Three-Factor Eating Questionnaires [17, 18]. In one respect, classical conditioning of appetitive reaction to actual or anticipated negative emotions appears to govern emotional eating behaviour [4]. Other facets of the emotional eating construct are potentially evident from correlates of the unidimensional measures of emotional eating. These include: reduced inhibitory control [19, 20]; reduced ability to differentiate internal bodily signals [16, 21]; perceived stress [22, 23]; intuitive eating [24]; and difficulties in emotion regulation [25, 26]. This evidence suggests that interventions should assess such ‘correlates’ to obtain accurate evaluation of outcomes. Despite the lack of predictive and discriminative validity of measures of emotional eating [2], a range of interventions have assessed its unidimensional nature because emotional eating impairs ability to make behaviour changes necessary for weight loss [27].

Interventions for weight loss have only touched the surface in terms of addressing emotional eating as a target outcome [28]. Behavioural interventions for weight loss have limited scope in reducing emotional eating [29] because they do not address issues specific to emotional eaters, namely their use of food to regulate emotions [28]. Mindfulness-based approaches have the potential to address this issue because they produce beneficial outcomes though modification of emotion regulation [30]. This is relevant to emotional eating as cross-sectional evidence indicates that experiential avoidance mediates the relationship between negative emotions and emotional eating [31]. Cross-sectional evidence also suggests that mindfulness is related to healthier eating, an association that may operate through greater acceptance connected to self-compassion [32]. Mindfulness meditation facilitates acceptance and tolerance of negative emotions [33]. Mindfulness-based interventions comprise educational advice and mindfulness meditation training, the latter adapted for a Western secular context from Eastern traditions of meditative contemplation, most notably, Buddhism [34]. Mindfulness can be described as a state of purposeful attention towards momentary experience holding an open, accepting and non-judging attitude [33, 35]. The most widely used mindfulness approach is the mindfulness-based stress reduction (MBSR) programme and its variants, originally devised by Kabat-Zinn [36]. Mindfulness meditation training (MMT) exerts its beneficial effects on a range of outcomes through attention regulation, emotion regulation (including reappraisal, exposure, extinction and reconsolidation), and enhanced executive functioning, most notably in terms of inhibitory control [37–42]. It is likely that the decentering quality of focused attention is a key mechanism by which reactivity to emotionally aversive and rewarding stimuli is reduced [43]. However, it is yet unclear which aspect of MBSR programmes, meditation or psychological education or their combination, account for beneficial effects [34].

Mindfulness-based interventions for weight loss have shown mixed success [28, 44–47]. Eating-related mindfulness interventions have mainly focused on weight and caloric intake as outcomes, rather than appetitive precursors of overeating [48]. The range of interventions used to date have varied in quality (lack of control groups), choice of primary outcomes (primarily weight and food intake), and curriculum configuration. Predominately the interventions have used modified MBSR and MBCT approaches and varied in the extent to which they embed a focus on eating behaviour [47]. Although a small number of these intervention studies have shown reduction in emotional eating scores, they have varied considerably in how they measured this construct. Emotional eating was neither a primary outcome measure nor a strong focus in intervention curricula [47]. An observational study of participants taking the MBSR programme for stress reduction demonstrated a clear reduction in emotional eating scores despite no focus on eating behaviour as part of the intervention [44], though the scale used to assess emotional eating was not validated. An evaluation of an uncontrolled smartphone delivered Mindful Eating programme demonstrated reduction in food craving connected to emotional eating in overweight and obese individuals [49]. A randomized controlled trial of mindfulness-based eating awareness training (MB-EAT) for those with threshold or sub-threshold binge eating disorder revealed a trend level reduction in emotional eating relative to controls [50]. A more comprehensive primary care trial of the MB-EAT intervention for people who are overweight or obese is in progress using a unidimensional measure of emotional eating as a primary outcome [51]. Although mindfulness has received increased attention as an addition to weight management programmes it remains unclear whether mindfulness actively influences weight loss [52]. It is more likely that mindfulness actively influences psychological dispositions associated with overeating and consequent weight gain.

The variability in the effects of mindfulness interventions for weight loss on emotional eating are likely to be partly due to restricted focus on measuring attitudinal aspects of emotional eating rather than a broader range of correlates of emotional eating, for example, cue-reactivity, inhibitory control, emotion regulation, and the ability to distinguish between hunger/satiety and emotional cues to eat. Weak inhibitory control and high reward sensitivity (manifesting as cue-reactivity) combine in some individuals prone to cue-reactive overeating and weight gain [53–55] and overeating in response to negative emotions [19]. Experimental studies indicate that enhancement of attention with a mindful attitude (taking a non-reactive and non-judgmental stance toward thoughts and emotions) can reduce food craving [56], chocolate consumption [57], and attenuate reactivity to rewarding food-cues [58, 59]. Research on the mechanisms by which mindfulness alters food-cue reactivity is ongoing, but it is likely to work by altering internal processes such as heightened awareness of hunger and satiety signals and decreasing the influence of emotional cues to eat [47]. The variation in outcomes observed in reviews suggest that mindfulness approaches should be tailored to the experience of emotional eating and its correlates to determine clearer evaluation of interventions [28, 46, 47]. Furthermore, because of the complexity of weight-loss interventions (e.g., mindfulness training dietary and exercise advice, goal setting, CBT), it is very difficult to determine if the mindfulness training per se influences outcomes [47, 52]. Arguably it may prove more effective to directly intervene to change a broader range of appetitive traits associated with emotional eating using an emotional eating specific mindfulness approach before embarking on weight loss attempts; in effect, re-calibrating one’s relationship with food by attenuating the disruptive effects of habitual emotional eating. Such an approach may better prepare individuals to engage more effectively with the complexity of establishing new dietary and physical activity habits necessary for weight loss.

The mindfulness-based programme designed for this study took such an approach, the sole aim being to address habitual appetitive reactions to emotions as precursors to overeating. Building upon work conducted by Kristeller et al. [50] and elements of the Mindful Eating programme [60], a mindfulness-based emotional eating awareness programme was designed. The taught element specifically focused on evidence-based content regarding cue-reactivity, impulse control, stress and the role of emotions in eating. Mindfulness meditation skills common to the MBSR approach were taught and tailored specifically to daily experience of eating behaviour and related emotions. The range of measures chosen reflected aspects of the curriculum taught on the course, and evidence regarding the correlates of emotional eating measures to date, namely: behavioural inhibition, emotion related impulse regulation, cue-driven eating, ability to distinguish between physical and emotional cues to eat (intuitive eating; see [47]), perceived stress, and a standard measure of emotional eating. Measures were administered before and after the 6-week course and change in their values assessed.

## Methods

### Participants and recruitment

Participants were recruited by advertising using emails, posters and flyers distributed throughout the campus of a North West UK University. 41 participants registered interest for the course; 23 enrolled and completed pre-course assessments. 18 attended the first class and 14 of those (age 29.4 years; 13 female) completed the course and the end-of-course assessments. Social media was also used to invite potential candidates. Recruitment messages and additional information specifically stated that the mindfulness course was “not a weight-loss intervention”; the following content was used in all advertising: “Are you affected by emotional eating? Would you like to change your emotional relationship with food and eating? If so, try the mindfulness-based emotional eating awareness training course.” Participants were invited to visit a dedicated website to read further information and register. Participants had to self-assess their eligibility using three inclusion and four exclusion criteria. The inclusion criteria were: (1) BMI between 18 and 29; and (2) history of problematic emotional eating; and (3) over 18 years of age. The exclusion criteria were: (1) current diagnosis for an eating disorder or other mental health condition; (2) food allergy or intolerance; (3) currently pregnant; and (4) experienced meditator. Participants in the BMI range from healthy to overweight (18–29 kg/m^2^) were invited to participate because emotional eating is not exclusive to individuals who are overweight. LJMU Research Ethics Committee Ethical provided approval for the research (ref: 15/NSP/018).

### Measures

#### Mindfulness

Dispositional mindfulness was assessed using the short-form of the Five-Facet Mindfulness Questionnaire—FFMQ-24 [61]. The FFMQ-24 comprises five subscales of which three are closely aligned with the widely used definition of mindfulness as “Paying attention in a particular way: on purpose, in the present moment, and nonjudgmentally [62]”. The FFMQ short-form has good internal consistency [61]. The current study used a composite of the non-reactivity, acting with awareness, and non-judging subscales. Previous use of the composite of these three subscales [63] indicates acceptable internal consistency (*α* = 0.79). The response format comprises a five-point Likert scale (1 = never or very rarely true; 5 = very often or always true). Scores are summed to produce totals for each subscale and a total scale score. Higher scores indicate higher levels of dispositional mindfulness.

#### Impulse control

One self-report and one behavioural measure of impulse control were used. One subscale of the Difficulties in Emotion Regulation Scale (DERS) [64] was administered, the impulse control difficulties scale (DERS-IC). Items on the DERS-IC are prefaced by the phrase, “When I’m upset…”, which reflects an approach focusing on emotional context that is distinct from a broader assessment of impulsiveness. In the development of the DERS, Gratz and Roemer [64] aligned their view of emotion regulation with theoretical positions [65, 66] that postulate adaptive emotion regulation to be “the ability to inhibit inappropriate or impulsive behaviours, and behave in accordance with desired goals when experiencing negative emotions” (p. 42). By using the DERS-IC we created the opportunity to examine impulse control as a facet of emotion regulation in accord with recent conceptualization of its presentation in emotional eating behaviour [5, 67, 68]. The stop-signal task was used as a behavioural measure of impulse control [69]. Performance on the task generates two key variables; signal reaction and stop delay. By subtracting the latter from signal reaction, the stop signal reaction time (SSRT) was derived for use in analysis. Longer SSRTs are indicative of decreased ability to inhibit responses when required.

#### Cue-driven and emotional eating

Two subscales of the 21-item revised Three-Factor Eating Questionnaire (TFEQ-R21) [18] were used to assess cue-driven eating (uncontrolled eating subscale: tendency to lose control over eating when feeling hungry or when exposed to food stimuli) and emotional eating (propensity to overeat in response to negative mood states). The TFEQ-R21 response format comprises a four-point response format (e.g., definitely true/mostly true/mostly false/definitely false). Scores are summed to produce scale scores and the raw scores are transformed to a 0–100 scale. Higher scores are indicative of greater cue-driven and emotional eating. Uncontrolled eating is indicative of heightened reactivity to external cues to eat. The TFEQ-R21 has acceptable internal consistency and criterion and discriminant validity [18].

#### Intuitive eating

The intuitive eating scale [70] measures tendency to follow physical hunger and satiety cues when determining when, what, and how much to eat. In this respect, it reflects ‘ability’ to distinguish between internal physical and emotional cues to eat. Two subscales were used in the current study: (1) eating for physical rather than emotional reasons, and (2) reliance on internal hunger and satiety cues to determine when and how much to eat. Responses to statements about tendencies (e.g., I stop eating when I feel full, not overstuffed) are made on a five-point Likert scale (5 = strongly agree, to 1 = strongly disagree). The scale has acceptable internal consistency (Cronbach’s *α* = 0.90).

#### Perceived stress

Stress was assessed using the Perceived Stress Scale (PSS) [71], which comprises ten statements about feelings and thoughts experienced during the last month, responded to on a five-point Likert scale (0 = never, almost never, sometimes, fairly often, and 4 = very often). For example: “In the last month, how often have you been upset because of something that happened unexpectedly?” The PSS has acceptable internal consistency (*α* = 0.85) and well-established validity [71]. Higher scores indicate higher levels of perceived stress.

### Intervention

The intervention was called mindfulness-based emotional eating awareness training, or Mbeeat, for short. A summary of the course curriculum is provided in Table 1. The design of the intervention incorporated psycho-educational content and training in mindfulness meditation integrated in weekly classes that delivered conceptual content and practical exercises. The mindfulness elements followed the content and structure typical of MBSR programmes [36], integrated in part with elements of the Mindful Eating course [60]. Three core mindfulness practices (body scan, breathing meditation, 3-min breathing space), and variations of them, were taught in class and discussed using inquiry approaches typical of MBSR programmes. Each week, these practices were also made specific to emotional eating contexts using methods from the Mindful Eating course [60]. The psycho-educational element was designed based on extant literature regarding the relationship between emotions and eating, stress and eating, and impulse control and reward motivated eating. Participants received a workbook for each class and guided meditation audio for use at home.

**Table 1:** Outline of Mbeeat course curriculum

Week 1: mindfulness and emotional eating	Concepts: mindfulness explained; emotions and mindful eating concepts
	Practice and inquiry: first-taste raisin task; guided breath meditation; 3-min breathing space
	Home practice: breath meditation, daily mindful activity, diary work, mindful eating exercises
Week 2: automatic reactions to emotions	Concepts: physical vs. emotional triggers of hunger; impulses, automatic reactivity, reward and the brain; introduction to acceptance
	Practice and inquiry: 7 kinds of hunger task [60]; identifying emotional needs; guided breath and guided body scan meditation
	Home practice: breath and body scan; 7 kinds of hunger task; daily mindful activity; diary work; noticing and savouring pleasant eating experiences
Week 3: stress and habitual reactions	Concepts: the stress cycle; habitual automatic reactions; reactive patterns of eating; becoming aware of cravings
	Practice and inquiry: identifying sources of craving task; guided breath meditation; being present with pleasant experiences task
	Home practice: body scan and breath meditation; focus on awareness of cravings and reaction vs. response; daily mindful activity; seeking out the pleasant and savouring the experience
Week 4: responding vs. reacting	Concepts: internal sensations and bodily cues related to emotional eating; responding rather than reacting to disturbing emotions; acceptance and the mindfulness paradox
	Practice and inquiry: breath meditation; accepting a difficult emotion practice specific to reactive eating patterns
	Home practice: guidance for ‘home retreat’ activities for week 5
Week 5: no class—‘home retreat’	Half-day committed to quiet time without media, phone, people, work, study, etc. Direction to set this time aside by careful planning and communication with relevant people to inform of this activity. Focus was on short mindfulness meditation practices and simple mindful activities such as household chores. Emphasis on mindful eating practice using learning to date. Direct attention to noticing the pleasant and unpleasant of the experience and practising acceptance rather than reaction
Week 6: review and next steps	Review of ‘retreat’ time experience. Six steps for mindful eating [60]. Noticing the inner critic and practice of loving-kindness meditation. Review of key concepts of course

### Procedure

An accredited mindfulness practitioner (Lattimore) delivered the course. The practitioner has over 8 years’ regular meditation experience, was trained to deliver mindfulness courses by Breathworks, and complies with the UK Network for Mindfulness-Based Teacher Training good practice guidelines. The intervention period lasted 8-week inclusive of pre- and post-intervention assessment. Over six consecutive weeks classes were delivered for 2.5 h per week. One week prior to the intervention start date, participants who had enrolled completed questionnaire measures and informed consent online, and attended laboratory assessment to complete the stop-signal task. Following completion of the course, participants returned to repeat the stop-signal task, repeated the questionnaires again, and were debriefed about the purpose of the research. Given the small sample size, distributions were checked using box-and-whisker plots and histograms for outliers and skew/kurtosis. Three cases were identified as anomalies on the SSRT measure as RTs were below what would be expected as realistic. These scores were removed prior to analysis leaving 11 cases for analysis of this variable. All questionnaire measures were normally distributed thus all 14 cases were used in analysis. Paired *t* tests and associated effect size *d*_rm_ [72] were calculated along with 95% confidence intervals for *d*_rm_ using Hedges’ *g* average [73]. Cohen’s *d*_rm_ was calculated based on *r, t* and *n* values. The false detection method [74] was used to adjust for family wise error rate (FDR rate = 20%).

## Results

Paired sample *t* tests were conducted to test differences in measures between baseline and end of intervention. Table 2 displays relevant means and associated statistics. There were significant reductions in stress and cue-driven eating and improvements in behavioural and self-reported impulse control. This implies that by the end of intervention, participants were experiencing less stress and less prone to reward motivated eating and impulsive behaviour. Regarding intuitive eating measures, participants reported stronger tendency to respond to internal hunger and satiety signals and a stronger tendency to respond to physical sensations rather than emotional cues to eat. This implies that that by the end of intervention, participants eating behaviour was more in-tune with internal hunger/satiety signals than external or emotional cues to eat. Although the difference in emotional eating scores showed a reduction in the tendency to eat for emotional reasons, the reduction was not significant. Mindfulness scores did not differ from baseline to end of intervention. All effect sizes were in the moderate to large range even after correction for potential sample size bias using the Hedges’ g metric.

**Table 2:** Study variables (M/SD) at baseline, end of intervention, and associated inferential statistics

	Baseline		End		*t*(14)^a^	*r*	*p*^b^	*d*_rm_	*g*	*g* 95% CI LL–UL
	*M*	*SD*	*M*	*SD*						
Perceived stress	19.6	5.5	13.2	6.3	4.94	0.32	0.005	1.54	1.01	0.33 to 1.80
Intuitive (hunger/satiety)	2.7	1.0	3.8	1.0	4.30	0.53	0.005	1.11	− 1.03	− 0.42 to − 1.74
Emotional impulse (DERS-IC)	15.1	2.9	12.4	2.2	3.41	0.39	0.005	1.00	0.98	0.32 to 1.74
Intuitive (physical/emotional)	2.6	0.9	3.5	0.8	3.40	0.32	0.005	1.06	− 2.86	− 0.31 to − 1.75
Cue-driven eating (TFEQ-UE)	58.7	23.2	45.0	24.4	2.68	0.68	0.025	0.58	0.54	0.09 to 1.04
Behavioural impulse (ms)	275.3	29.1	238.4	44.9	2.58	0.37	0.050	0.87	0.90	0.15 to 1.75
Emotional eating (TFEQ-EE)	54.8	23.1	40.1	20.7	2.03	0.24	0.075	0.66	0.63	− 0.03 to 1.35
Mindfulness	41.6	7.7	44.9	4.9	1.30	0.04	0.100	0.17	− 0.48	− 1.25 to 0.25

^a^*df* = 11; *d* = Cohen’s *d*_rm_ effect size; *g* = Hedges’ *g*

^b^*α* corrected for familywise error rate using false detection method

## Discussion

Emotional eating is a construct that arguably requires refinement to reflect its multifaceted nature [4] so that weight-loss interventions can accurately assess the impact of emotional eating on outcomes [10, 27]. The aim of the present study was to assess the unidimensional aspect of emotional eating to determine how a mindfulness-based intervention may attenuate emotional eating and its potential correlates: impulse control, cue-reactivity, ability to discriminate between physical and emotional cues to eat (intuitive eating), and stress. The intervention purposefully did not focus on weight loss and recruited participants who had self-declared difficulties with emotional eating.

The effect size estimates and associated confidence intervals indicate a strong effect for change in intuitive eating scores (2.86 SD units), indicating that participants report greater reliance on physical rather than emotional cues to eat at end of intervention. At the end of intervention, there was a moderate reduction in perceived stress and an increased reliance on hunger/satiety cues (1 SD unit). Additionally, a reduced tendency to act impulsively in response to negative emotions and improved response inhibition (1 SD approx.) were observed. A weaker effect for cue-driven eating (0.5 SD unit) was evident indicating a reduction in tendency to be reactive to external food-cues by the end of intervention. Although similar a reduction (0.5 SD unit approx.) for emotional eating and increase for mindfulness scores was observed, the confidence intervals for these effects and adjusted significance levels suggest that the sample was not large enough to draw reliable conclusions about the effect of the intervention on these two measures. The distinctive feature of the intervention was a focus on an array of psychological factors related to emotional eating and not on weight loss or dietary change. Without an active control, it is not possible to determine which element of the intervention influenced change in parameters measured. It is possible that the meditation practices and/or the psycho-educational advice caused the changes observed. Mindfulness meditation can produce effective attention regulation, emotion regulation, and enhanced executive function [37–42]. It is plausible to assume that the MBeeat intervention created the conditions that enabled participants to re-calibrate their relationship with food using mindfulness. In effect they became more “in-tune” with or aware of the influence of external and internal triggers to eat, obtained distance from the influence of aversive emotions, in effect becoming less reactive, and developed enhanced control over impulsive urges. This explanation can be supported in part by the evidence reviewed regarding the positive effects of mindfulness on varied measures of cue-driven and emotional eating [31, 32, 44, 49, 50, 56–59]. However, until a randomized trial is conducted to disentangle the meditation and educational elements compared to an active control such conclusions warrant cautious interpretation.

To date, mindfulness-based interventions for weight loss have indicated that adding a mindfulness component to weight-loss interventions has the potential to strengthen their effectiveness [44, 47, 48, 50, 52]. The current study presented a case for mindfulness training occurring before weight loss is attempted, to specifically address psychological traits that underpin overeating and undermine weight-loss efforts. The outcomes accord with the evidence that psychological traits associated with weight gain should be targets for intervention [28, 46–48]. Evidence is lacking regarding intervention prior to weight loss to address emotional eating and its correlates. The notable effect sizes in the current study suggest that emotional eating and its correlates can be modified using mindfulness techniques and thus may prepare individuals better for weight-loss intervention. Furthermore, the evidence that probable correlates of emotional eating change as a result of the Mbeeat intervention suggests that the psychological profile of emotional eaters requires further study rather than relying upon a unidimensional assessment of emotional eating [4]. Establishing a credible multi-faceted assessment of emotional eaters will aid precision is assessment of intervention outcomes.

## Conclusion

There are limitations to the evidence presented. Lack of an appropriate control group, self-selecting selection bias, and potential demand characteristics associated with self-report measures compromise the internal validity of the design. Notwithstanding these limitations, the outcome of this study provides proof of principle as a basis to design a randomized control trial to assess rigorously the effectiveness of the Mbeeat approach as a precursor to a weight-loss intervention. Furthermore, the outcomes suggest that by examining the wider dimensions of emotional eating in terms of its relation to stress, intuitive eating, inhibitory control and emotion regulation, research may discover if and how change in such factors operates synergistically.
